# How to Efficiently Produce Ultrapure Acids

**DOI:** 10.1155/2019/5180610

**Published:** 2019-01-01

**Authors:** Damiano Monticelli, Alessio Castelletti, Davide Civati, Sandro Recchia, Carlo Dossi

**Affiliations:** ^1^Dipartimento di Scienza e Alta Tecnologia, Università degli Studi dell'Insubria, Via Valleggio 11, 22100 Como, Italy; ^2^Dipartimento di Scienze Teoriche e Applicate, Università degli Studi dell'Insubria, Via J.H. Dunant 3, 21100 Varese, Italy

## Abstract

Subboiling distillation has been used since two decades for the purification of analytical grade acids from inorganic contaminants and demonstrated an efficient method to obtain pure acids starting from reagent grade chemicals. Nevertheless, the effect of the subboiling parameters on the purity of the distilled acids has never been methodically investigated. Aim of the present research is a systematic evaluation of the subboiling distillation protocol for the production of pure hydrochloric and nitric acid. In particular, the effect of the subboiling temperature and the number of subsequent distillations was investigated as these parameters were recognised as the most important factors controlling acid purity, acid concentration, and distillation yield. The concentration of twenty elements in the purified acids was determined by Inductively Coupled Plasma-Mass Spectrometry. As a result, the subboiling temperature (up to 82°C) and the number of subsequent distillations (up to four) were demonstrated not to affect the purity of the distilled nitric and hydrochloric acids. Under normal laboratory conditions, the residual elemental concentrations were in most cases below 10 ng/L in both nitric (2.75% w/w) and hydrochloric (0.1 M) blanks. Ultrapure nitric and hydrochloric acids could accordingly be produced under the most favorable conditions, i.e., the highest temperature and one distillation process only.

## 1. Introduction

Analytical chemists are increasingly being required to analyze samples having trace metal concentration at very low level, under nanomolar concentration. At these concentration levels, the contamination caused by sampling, storage, manipulation, and any added reagent is the key factor controlling the accuracy in trace element determination [1, 2]. In particular, high purity acids are regularly used for the dissolution and storage of samples, the cleaning of sample containers, standard solution preparation, and, in general, cleaning and conditioning of all the analytical equipment and instrumentation. Detection of element concentrations and isotopic pattern in ice cores [3–5], snow [6], and the water column [7] in the Antarctic continent is an excellent example of state-of-the-art requirements for high purity acids.

Ultrapure acids can be directly produced in the laboratory by subboiling systems: homemade production is almost mandatory when high quantities (25-100 mL/day) are needed [3, 8] as in a research laboratory. Subboiling distillation, which has been used since two decades [2, 9], is known to be an efficient system for the purification of analytical grade acids. The method is based on the heating (typically by IR irradiation) of the liquid under its boiling temperature [10]: the generated vapors are subsequently condensed on a cold finger and collected in a clean plastic bottle, usually made of fluorinated polymers.

Although subboiling distillation is commonly used for the purification process, a critical thorough study of the quality and yields of distillates as a function of the instrumental parameters (i.e., IR heating power and number of subsequent distillations) has never been performed. The literature reports a small number of papers dealing with the purity of the distilled acids and a limited optimization of the distillation process [3, 8–11]. Accordingly, we decided to systematically assess the performances of the distillation process, aiming at defining the best conditions assuring maximum yield and purity. The study focused on hydrochloric and nitric acids, which are commonly used in analytical laboratories and industrial processes [3, 8].

## 2. Experimental

### 2.1. Reagents and Chemicals

Ultrapure water from a Millipore Gradient A10 MilliQ system (18 MΩ•cm resistivity, <5 ppb TOC) was used throughout. Analytical grade nitric and hydrochloric acids from Carlo Erba Reagenti were used as the starting acids: declared concentrations are between 69.1 and 69.9 % w/w for HNO_3_ and ≥ 36.5 % w/w for HCl. Solution preparation and sample manipulation were executed in a class 100 laminar flow hood. Sample bottles (LDPE, low density polyethylene from Nalgene) were cleaned by soaking in a diluted detergent solution (Nalgene L900) and rinsed with ultrapure water followed by two cycles of five day soaking in 2% pure nitric acid and rinsing with ultrapure water.

### 2.2. Subboiling Apparatus and Optimization Procedure

A Milestone DuoPUR subboiling equipment was used for acid purification. The system features two independent quartz stills, so that HCl and HNO_3_ may be purified simultaneously. IR irradiation is used for heating, tap water for cooling and the distillate is collected in a PFA bottle.

The effect of subboiling temperature and number of subsequent subboiling procedures was investigated systematically in a full factorial experimental design. Four levels of temperature (56, 65, 73, and 82°C, corresponding to power settings of 20%, 30%, 40%, and 50%) and four subsequent distillations were tested, totaling sixteen experiments. Temperatures higher than 82°C lead to a visible decomposition of nitric acid and were accordingly not employed. Four acid aliquots were collected for each experiment in four different bottles to evaluate interbottle variations. Acid aliquots were directly collected from the distillation bottle after cooling by an acid resistant, repetitive pipette (Eppendorf Repeater Plus) after thorough rinsing of the pipette tip. Acid aliquots were subsequently diluted with ultrapure water to 2.75% by weight for nitric acid and to 0.1 M for hydrochloric acid. The hourly yield and acid concentration in the distillate were also determined: the latter was evaluated after each distillation step and for each temperature by titration with standard sodium hydroxide.

The effect of the factors (subboiling temperature and number of subsequent distillations) was assessed by Analysis of Variance (ANOVA). For each element, the within group variance (replicated determinations) was compared with the treatments' variance, i.e., subsequent distillations and distillation temperature. A significance level of 0.05 was employed.

### 2.3. Trace Element Quantification

Trace metals were determined by Inductively Coupled Plasma-Mass Spectrometry (Thermo Elemental, mod. X-Series^II^). Optimization of the instrumental parameters was performed daily as recommended by the manufacturer with a 1 *μ*g/L multistandard solution, whereas the mass calibration of the quadrupole was verified weekly. As a result, low levels of oxides (CeO^+^/Ce^+^<2%) and double charged ions (Ba^++^/Ba^+^<3%) were achieved. Because of the low investigated level of elements, the major contribution to uncertainty in the determination is due to the reproducibility of the signals: the percentage relative standard deviation (RSD%)is between 30% and 20% for concentrations in the range 0.1 to 1 ng/L, between 10%and 20%for concentrations ranging from 1 to 10 ng/L and below 10% thereafter.

Trace element concentrations were determined on diluted solutions of nitric and hydrochloric acid: concentrations close to the maximum recommended by the instrument manufacturer were used (2.75% nitric and 0.1 M hydrochloric acid).

The standard solutions used for external calibration were prepared daily by serial dilution of concentrated standards obtained from single element standard solutions (Fluka). Final concentrations were between 0.1 and 100 ng/L, depending on the analyte. The following isotopes were measured: 27Al, 107Ag, 137Ba, 9Be, 209Bi, 111Cd, 59Co, 65Cu, 133Cs, 69Ga, 115In, 7Li, 24Mg, 55Mn, 60Ni, 208Pb, 195Pt, 88Sr, 205Tl, 51V (in nitric acid only), 238U, 66Zn. The choice of the isotope is a compromise between maximum abundance and absence of isotopic interferences. Vanadium could not be determined in hydrochloric acids because of the well-known 51ClO polyatomic interference. The level of other elements, i.e., Hg, Cr and Se, could not be determined as lower than the detection limits.

## 3. Results and Discussion

The general characteristics of the distillates will be discussed first, whereas the effect of the distillation parameters on acid purity will be presented in the following sections.

### 3.1. Production Yields

The production yields for the two acids were similar and increased linearly with temperature. In particular, the production yield doubled for both nitric acid (from 11 to 22 mL/h) and hydrochloric acid (from 13 to 25 mL/h) when the subboiling temperature was raised from 56 to 82°C. These data refer to a starting volume of 100 mL: the use of larger volumes (e.g., 500 mL) lead to higher productivities as the evaporating surface was consistently increased (around 75 mL/h for 500 mL). A subboiling temperature around 80 degrees is therefore recommended as a good compromise between productivity and limited degradation of nitric acid: moreover, increasing the volume of acid in the boiling still lead to a fourfold increase in acid yield.

### 3.2. Acid Concentration in the Distillates

The concentration of the distilled acids was determined to assess whether the temperature of the subboiling distillation or the subsequent distillations could affect their concentrations.

Nitric acid showed a decrease in concentration as a function of both subboiling temperature and subsequent distillations. The temperature had the highest effect, lowering the concentration from 68% w/w to 64% w/w, when the temperature was raised to 82°C (average of the four subsequent distillations). Performing four subsequent distillations had a limited effect, reducing the acid concentration by around 1% w/w for 56 and 65 degrees, whereas no effect was observed when the temperature was raised to 73 and 82°C. This reduction was due to the limited thermal decomposition of nitric acid with increasing temperature, standing the starting 68% w/w concentration is the azeotrope for the mixture HNO_3_/H_2_O.

Similar trends were observed for hydrochloric acid: a constant decrease in concentration with increasing temperature and number of distillation was evidenced. The concentration was reduced to 31% in the worst case, i.e., when the highest temperature and four distillation steps were used (see also Anil et al. [10]). The reduction in HCl concentration is possibly due to the acid partially escaping through the vapor ventilation system (the boiling still is not sealed; a vent system ensures that no overpressure is generated).

### 3.3. Effect of Subsequent Distillations

**Figure 1:**
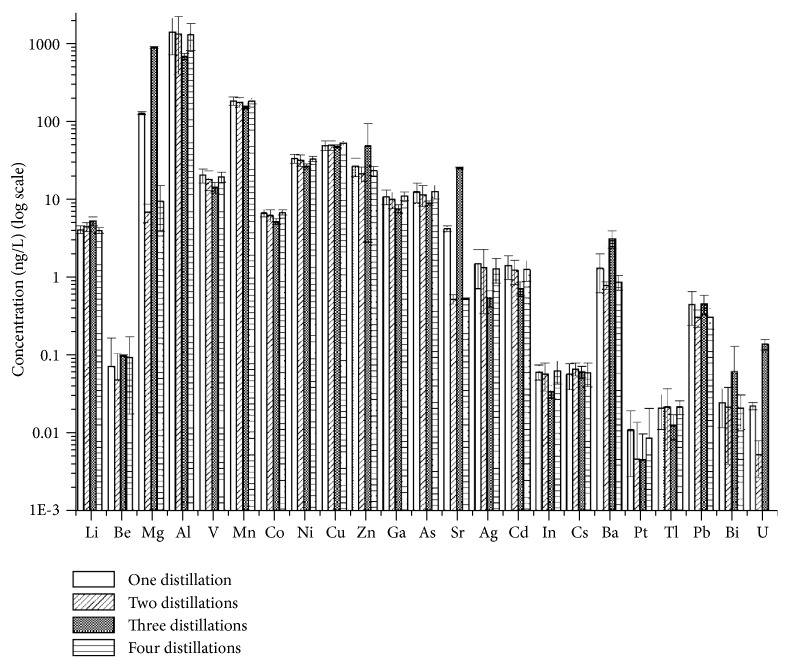
Effect of subsequent distillations on trace element concentrations in HNO_3_ (subboiling temperature: 82°C). Error bars correspond to ±1 standard deviation based on the four analyzed aliquots.

The effect of subsequent distillations on acid purity was systematically evaluated by distilling the same acid aliquot four times. For each subboiling temperature, the results showed that the variance of trace element concentration due to the four distillations is not distinguishable from the within group, i.e., four sample aliquots, variance (ANOVA test, significance level 0.05). The results for all the investigated elements were reported for nitric acid in Figure 1 for the highest investigated temperature (82°C).

The same behavior was observed for both analyzed acids. This feature may be explained by two different reasons. Firstly, the acid is directly inserted into the quartz still after each distillation, i.e., the still is not cleaned and/or rinsed between subsequent distillations. Solid deposits left by the previous distillation could be thus redissolved in the newly introduced acid leading to no benefit in subsequent distillation steps. Nevertheless, it cannot be ruled out that the obtained concentrations are environment limited, i.e., that they represent the lowest achievable limits in the laboratory environment.

### 3.4. Effect of Subboiling Temperature

**Figure 2:**
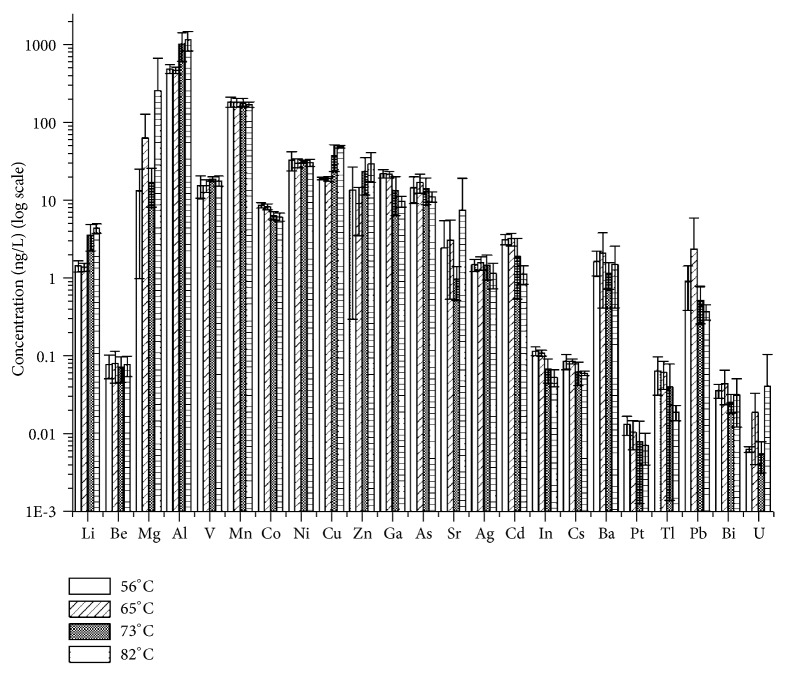
Effect of the subboiling temperature on trace element concentrations in HNO_3_ (one distillation). Error bars correspond to ±1 standard deviation based on the four analyzed aliquots.

The effect of the subboiling temperature was systematically investigated in a similar way as the effect of the subsequent distillations.  Figure 2 depicts the results obtained for nitric acid.

Trace element concentrations in nitric acid were shown not to depend on subboiling temperature (ANOVA test). Opposite to this trend, the concentrations of lithium, aluminum, copper, and cadmium were statistically different among the four temperature (ANOVA test, p<0.01). In particular, an increase of concentration with temperature was observed for the first three elements, whereas cadmium decreased with the temperature.

Regarding hydrochloric acid, no effect of the temperature was observed, besides lithium, zinc, and silver (ANOVA test, significance p<0.04) that showed lower concentrations at higher temperature.

The absence of any trend between distillation temperature and concentrations for most of the elements is due to the absence of volatile forms of the elements under the used experimental conditions. Understanding the significant trends that a limited number of elements show as a function of temperature (see the previous paragraphs) is a difficult task. Nevertheless, standing the very low involved concentrations, a limited number of outliers may be expected.

### 3.5. Acid Purity under Optimized Conditions

The preceding sections showed that the optimal conditions for subboiling distillation, i.e., the ones ensuring the highest purity and fastest process, include a single distillation step and a temperature of 82°C. In general, the elements in the prepared blanks (2.75% HNO_3_ and 0.1 M HCl) showed very low concentrations, among tenth and tens of ng/L, beside Al and Mn in nitric acid (see Table 1). This result confirmed the high efficiency of the subboiling distillation for acid purification: the purification efficiency, i.e., the ratio of the concentrations in the purified and unpurified acid, varied between <1% and 92% (median 11%). A few exceptions were observed, namely, Ga, Co, and Cd in nitric acid which increased their concentrations after distillation. The latter feature is possibly due to environmental contamination.

## 4. Conclusions

The subboiling distillation protocol for the production of pure hydrochloric and nitric acid was systematically evaluated. In particular, it was shown that, under the experimental conditions normally employed, the number of subsequent distillation cycles and the temperature of the subboiling process (up to 82°C) did not affect the acid purity.

Regarding yields, the comparison with literature data showed that similar acid yields are obtained, despite the differences in lamp powers, pointing out that the still design and efficiency are the main factors controlling acid yields (e.g., lamps with powers of 1300 and 80 W resulted in only a fourfold increase in yields for HCl production [9, 10]).

Comparison of contamination levels after purification with literature data to obtain indications on the best process/instrumentation/manipulation is not easy as trace element concentrations determined in purified nitric and hydrochloric acids vary notably in the literature [2, 3, 8–11]. Both higher and lower levels than the ones observed in the present study are reported, evidencing that the design of the apparatus and/or the adopted procedures strongly influence the outcome of the subboiling process. Nevertheless, the present study showed for the first time that the effect of the distillation conditions (temperature and number of subsequent distillations) is not distinguishable from the variance due to the experimental errors in the determination of the elements. Accordingly, this study demonstrates that ultrapure nitric and hydrochloric acids can be produced under the most favorable conditions, i.e., the highest temperature and one distillation cycle only, without compromising the quality of the distillates.

## Figures and Tables

**Table 1:** Elemental concentrations (C) in blank solutions under optimized conditions (subboiling temperature 82°C, one distillation cycle).

	C > 100 ng/L	100 < C < 10 ng/L	10 < C < 1 ng/L	C < 1 ng/L
Nitric acid blank 2.75% w/w	Al Mn	Cu Ga Mg Ni V Zn	Ag Ba Cd Co Li Sr	Be Bi Cs In Pb Pt Tl U

Hydrochloric acid blank 0.1 M	--	Al Mg Mn Ni Zn	Cu Ga Li	Ag Ba Bi Cd Co Cs In Pb Pt Sr Tl U

## Data Availability

The numerical data used to support the findings of this study are included within the article and there is no restriction on data access.
